# Dynamic Self‐Optimization of PtNi Nanocatalysts Through the Concurrent Evolution of Active Sites and Interfacial Environment During Reaction

**DOI:** 10.1002/advs.78749

**Published:** 2026-11-11

**Authors:** Weizhi Wang, Yingying Cai, Pengfei Tian, Jing Xu, Mingliang Zhu, Fuzhen Xuan

**Affiliations:** ^1^ School of Chemical Engineering East China University of Science and Technology Shanghai China; ^2^ Shanghai Key Laboratory of Intelligent Sensing and Detection Technology School of Mechanical and Power Engineering East China University of Science and Technology Shanghai China

**Keywords:** dynamic structure, heterogeneous catalysis, in situ characterizations, interfacial water, oxygen reduction reaction, reconstruction

## Abstract

Heterogeneous catalysis is shifting from static material design to steering dynamic systems. Here, using the oxygen reduction reaction (ORR) over PtNi catalysts as a model system, we show that the leaching and structural reconstruction of PtNi alloys during the reaction do not degrade performance but actively drive the catalyst toward a superior state. Combining quasi‐in situ transmission electron microscopy (TEM), quasi‐in situ x‐ray diffraction (XRD), in situ attenuated total reflection Fourier transform infrared (ATR‐FTIR) spectroscopy, and theoretical calculations, we decipher a potential‐driven reconstruction process, initiated by Ni leaching and Pt dissolution‐reposition, that transforms initial polyhedral core‐shell structures into highly active nanocages. This yields a 2.3‐fold enhancement in mass activity at 0.9 V vs. RHE through two synergistic factors. First, the newly formed porous structure increases the population of highly active, low‐coordination Pt sites for O_2_ activation. Second, the restructured surface templates the interfacial water into a network featuring stronger symmetric hydrogen bonds, consistent with a more favorable interfacial environment for proton transfer. This concerted optimization of both the solid catalyst and the liquid environment during reaction process extends the static paradigm of catalyst design and introduces dynamic interfacial water engineering as a vital dimension for advancing electrocatalyst development.

## Graphical abstract

Using quasi‐in situ TEM/XRD and in situ ATR‐FTIR spectroscopy, we reveal a potential‐driven self‐optimization mechanism in PtNi for ORR via dynamic reconstruction during reaction. Ni leaching and Pt redeposition transform the core‐shell structure into a nanocage, boosting activity 2.3‐fold. The enhancement arises from synergistic effects: more highly active, low‐coordination Pt sites and a strengthened interfacial hydrogen‐bond network.

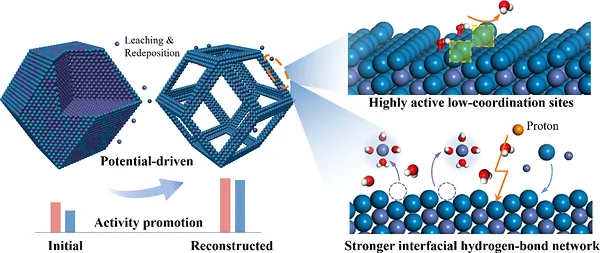

## 1 Introduction

Catalyst design principles based on established structural and electronic descriptors, including composition, local coordination, strain, and ligand effects, have provided a foundational framework for developing highly active electrocatalysts [1, 2, 3]. As the field advances, extending this static paradigm to incorporate a dynamic perspective has become increasingly vital [4, 5, 6, 7, 8, 9, 10]. Although rationally designed initial structures remain essential, electrocatalysts frequently undergo compositional, structural, and interfacial transformations under reaction conditions [11, 12, 13, 14, 15, 16]. Accounting for these dynamic processes therefore builds upon classical structure–activity principles and provides a more comprehensive basis for understanding and optimizing catalytic performance [17, 18, 19].

Extensive research has demonstrated that reconstruction can generate catalytically favorable sites in electrochemical reactions such as the oxygen evolution reaction (OER) [20, 21, 22, 23], hydrogen evolution reaction (HER) [24, 25], and CO_2_ reduction reaction (CO_2_RR) [26, 27], where the active structures frequently undergo changes in phase, coordination, or oxidation state [28]. In Pt‐based alloy catalysts for the oxygen reduction reaction (ORR), however, structural evolution has predominantly been associated with transition‐metal dissolution, particle growth, and catalytic degradation [29, 30, 31, 32, 33, 34, 35, 36]. At the same time, controlled dealloying has been successfully employed to create highly active Pt‐alloy nanocages, nanoframes, and nanofoams. Previous studies have primarily related the enhanced ORR performance of these porous structures to their final morphology and solid‐state properties, including increased surface area, Pt‐rich surfaces, residual strain, and exposed facets [37, 38]. By comparison, the concurrent response of the dynamic solid–liquid interface during catalyst reconstruction has received considerably less attention [39].

Electrocatalysis occurs at the complex solid‐liquid interface. During the ORR, the organization of interfacial water molecules and their associated hydrogen‐bonding networks can influence reaction‐intermediate stabilization, proton‐coupled electron transfer, and reaction kinetics [40, 41, 42, 43, 44, 45]. Although the structural evolution of Pt‐alloy nanoparticles into porous or hollow architectures has been extensively investigated, how this morphological reconstruction is coupled with the dynamic reorganization of interfacial water remains insufficiently understood. Establishing this connection is therefore important for understanding cycling‐induced activation beyond changes in catalyst morphology alone.

Herein, we employ structurally well‐defined PtNi nanocatalysts as a model platform to investigate the coupled evolution of active surfaces and interfacial water during potential cycling. By combining quasi‐in situ transmission electron microscopy (TEM) and x‐ray diffraction (XRD), in situ attenuated total reflection Fourier transform infrared (ATR‐FTIR) spectroscopy, density functional theory calculations, and explicit‐water ab initio molecular dynamics simulations, we follow the progressive evolution of catalyst morphology and interfacial environment. The activity enhancement is accompanied by the transformation of the initial polyhedral particles into Pt‐rich nanocages and the simultaneous reorganization of the interfacial hydrogen‐bond network. In particular, the reconstructed surface exhibits more favorable ORR energetics and supports a more connected and geometrically compact interfacial water network. These findings reveal a dynamic self‐optimization process involving the concurrent evolution of the catalyst surface and its surrounding solvent environment, thereby highlighting the importance of incorporating interfacial‐water dynamics into the design and mechanistic understanding of electrocatalysts.

## 2 Results and Discussion

### 2.1 Initial Structure and the ORR Performance

The TEM and high‐angle annular dark‐field scanning transmission electron microscopy (HAADF‐STEM) micrographs (Figure 1a,b) show a homogeneous dispersion of polyhedral PtNi nanoparticles with an average size of approximately 50 nm on the carbon support. Energy‐dispersive x‐ray spectroscopy (EDS) elemental mapping (Figure 1c–e) reveals that Ni is predominantly located in the core, while Pt is enriched at the surface. This core‐shell configuration is quantitatively confirmed by EDS line scan across an individual nanoparticle (Figure 1g–i): the Ni signal reaches its maximum at the particle center and decreases toward the edges, whereas the Pt signal is strongest at the surface. Figure 1f displays distinct diffraction peaks at 44.4°, 51.7°, and 76.1°, which match well with the (111), (200), and (220) planes, respectively, of a face‐centered cubic (fcc) Ni crystal lattice. Notably, these peaks exhibit an asymmetry shift toward lower 2θ values, indicating the presence of a segregated Pt‐rich phase alongside the primary Ni‐rich phase [46]. This XRD‐based conclusion corroborates the core‐shell structure directly observed by TEM and XPS (Figure S1).

**FIGURE 1:**
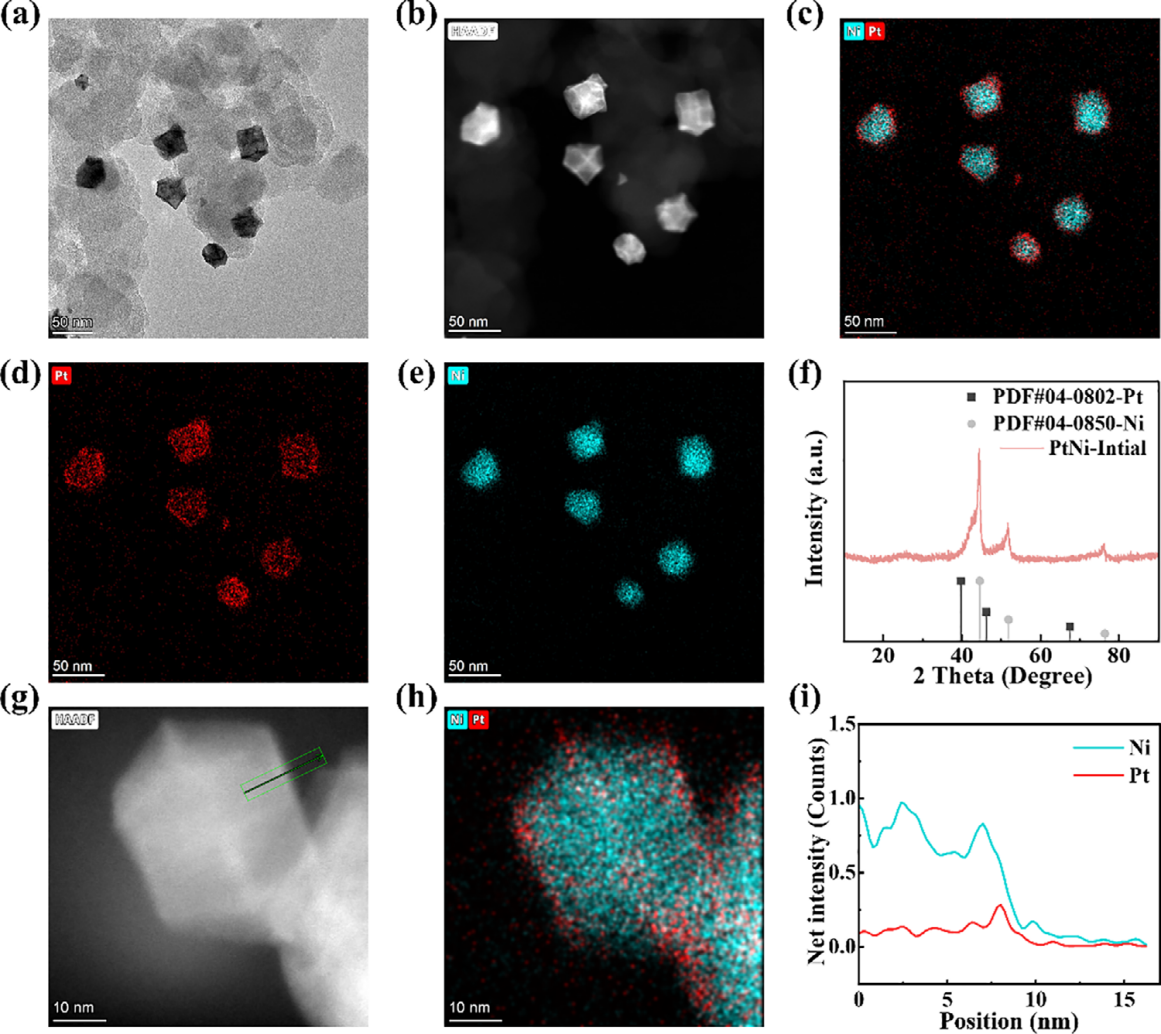
Initial structural characterization of the PtNi catalyst. (a) TEM image. (b) HAADF‐STEM image. (c–e) EDS elemental mapping. (f) XRD pattern. (g) HAADF‐STEM image of the line‐scan region. (h) Corresponding Ni and Pt elemental distribution of the line‐scan region. (i) EDS Line‐scan profiles of Ni and Pt across the selected region.

The ORR performance results are presented in Figure 2, Figure S2, and Table S1. Electrochemical cycling induced a pronounced performance enhancement in the PtNi catalyst. Figure 2a shows the positive shift of the cyclic voltammetry (CV) reduction peak from its initial 0.632 to 0.737 V after 1000 cycles, followed by its subsequent stabilization at ∼0.800 V after 10 000 cycles. The half‐wave potential (E_1/2_), derived from the linear sweep voltammograms (LSV) (Figure 2d), also increased from 0.907 V to a maximum of 0.924 V. Both metrics converged to stable values after 10 000 cycles (Figure 2d), indicating the completion of the activation process. Additional measurements of PtNi catalysts with different initial Pt/Ni ratios showed positive shifts in E_1/2_ for all compositions during cycling, with increases of 14–21 mV after 10k cycles (Figure S3), indicating that cycling‐induced activation is common across this PtNi series, while its magnitude depends on the initial composition.

**FIGURE 2:**
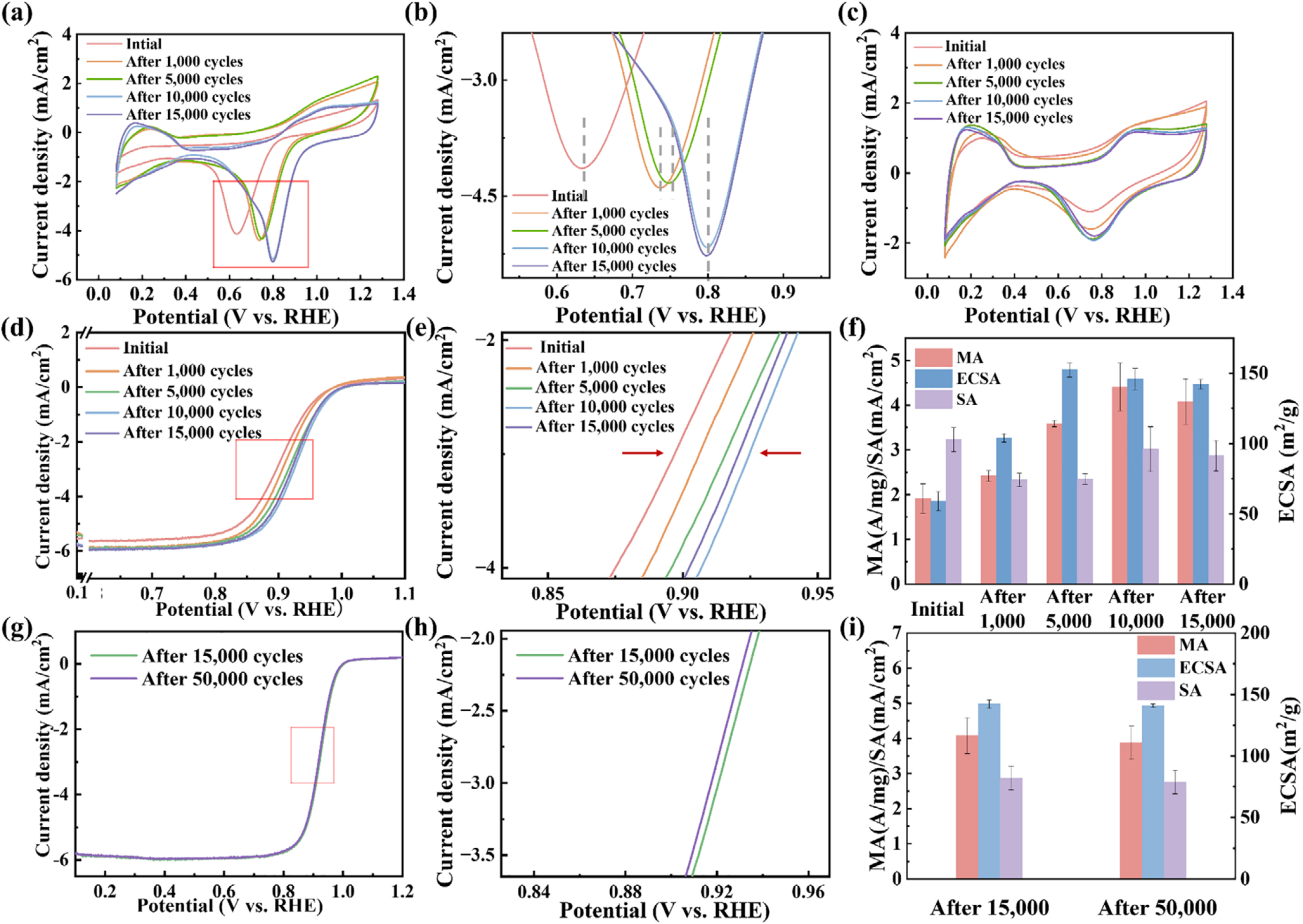
(a) CV curves of PtNi in O_2_‐saturated 0.1 M HClO_4_ solution after different numbers of CV cycles (scan rate: 50 mV/s), and (b) the corresponding magnified view of the reduction peaks. (c) CV curves of PtNi in Ar‐saturated 0.1 M HClO_4_ solution after different CV cycles. (d) ORR polarization curves of PtNi after different CV cycles (rotation speed: 1600 rpm, scan rate: 10 mV/s), and (e) the magnified view of the half‐wave potential region. (f) Evolution of MA, ECSA, and SA of PtNi as a function of CV cycle number. (g) ORR polarization curves of PtNi after 15 000 and 50 000 cycles (rotation speed: 1600 rpm, scan rate: 10 mV/s), and (h) the magnified view of the half‐wave potential region. (i) Comparison of MA, ECSA, and SA of PtNi after 15 000 and after 50 000 cycles. All data in (f) and (i) are presented as mean ± SD (*n* = 3).

Insight into the activation process was obtained from the evolution of the electrochemical active surface area (ECSA), which increased from 59.01 to 103.87 m^2^/g with the first 1000 cycles, followed by a pronounced rise to 152.45 m^2^/g after 5000 cycles, indicating substantial reconstruction of the catalyst surface that exposed more active sites (Figure 2f). Subsequently, after 15 000 cycles, the ECSA experienced only a minor decrease to 142.33 m^2^/g, demonstrating remarkable structural robustness. The mass activity (MA) exhibited a similar trend, increasing from 1.91 A/mg_Pt_ to a maximum of 4.41 A/mg_Pt_ after 10 000 cycles. Conversely, the specific activity (SA) initially decreased due to the rapid expansion of the ECSA, but later recovered and stabilized as the surface structure was optimized. Notably, during the 5000 to 10 000 cycles period, the ECSA showed a slight decrease while both MA and SA increased synchronously (Figure 2f). This divergence among MA, SA, and ECSA underscores that the performance enhancement is not solely attributable to an increase in the number of active sites, but also to an improvement in the intrinsic activity of each site. This improvement likely arises from the optimized surface composition and coordination environment formed during reconstruction. Most importantly, the catalyst exhibited exceptional long‐term stability. After an accelerated durability test (ADT) of 50 000 cycles, the degradation in half‐wave potential (E_1/2_) was negligible (Figure 2g), and the SA decreased by only 3.8% (from 2.87 to 2.76 mA/cm^2^), with the MA and ECSA retention rates reaching 95.1% and 99.0%—a performance surpassing most reported PtNi‐based catalysts and Pt‐based nanocage catalysts (Tables S2 and S3) [47, 48, 49, 50]. This exceptional stability attests to the durability of the in situ reconstructed surface structure under operating conditions.

### 2.2 Structural Evolution of the PtNi Catalyst During Reaction

Quasi‐in‐situ TEM was employed to track the structural evolution of the PtNi during CV activation, revealing the origin of its enhanced performance. Initially, PtNi exhibits a Ni‐core/Pt‐shell structure (Figure 3a,c and Figure S4), consistent with its state prior to sample preparation. After 1000 cycles, EDS analysis indicates an increase in the atomic fraction of Pt from 6.4% to 14.4%. As shown in Figure 3b and Figure S5, Ni remains predominantly in the core region, but the Pt signal is enhanced compared to the initial state, as confirmed by line‐scan analysis (Figure 3h–j). More importantly, the particles begin to exhibit irregular morphology with blurred boundaries, and the Pt signal is more intense at the edges and corners (skeletal regions) than on the flat facets. This observation suggests selective corrosion of the surface Pt(111) facets, which subsequently leads to the dissolution of subsurface Ni [30, 34, 35]. This initial corrosion and dissolution process lays the groundwork for the subsequent structural reconstruction. As the cycling proceeded to 5000 cycles, the compositional evolution intensifies, with the atomic fraction of Ni dropping sharply from 93.6% to 45.6%. Correspondingly, the signal intensity and size of the Ni core diminish significantly, while the Pt‐rich framework becomes more distinct (Figures 3d,f,g and S6), showing pronounced enrichment at the edges and corners. This indicates concurrent processes of continued Ni leaching and Pt dissolution‐redeposition, with the redeposited Pt preferentially stabilizing at high‐surface‐energy skeletal sites. Ultimately, after 10 000 cycles, the catalyst underwent a remarkable structural transformation, forming a stable PtNi alloy nanocage structure (Figure 3e and Figure S7). And after 50 000 cycles, the hollow nanocage morphology is well retained with no obvious particle coarsening or framework collapse (Figure S8).

**FIGURE 3:**
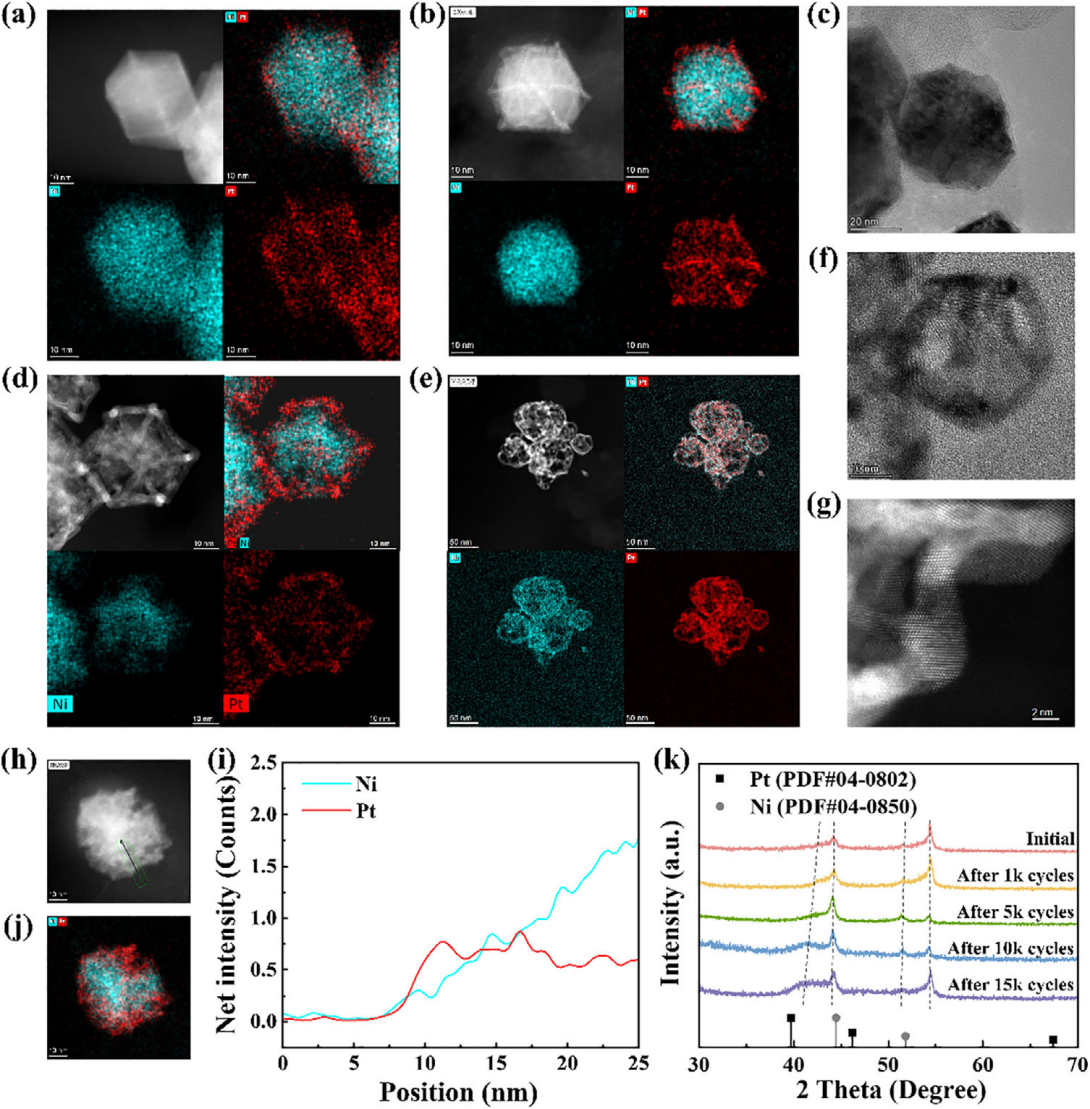
HAADF‐STEM images and corresponding EDS elemental mapping of PtNi at different stages: (a) initial, (b) after 1000 cycles, (d) after 5000 cycles, and (e) after 10 000 cycles. (c) TEM image of PtNi at the initial, and (f) after 10 000 cycles. (g) HAADF‐STEM image of PtNi after 10 000 cycles. (h) HAADF‐STEM image of PtNi after 1000 cycles, with the arrow indicating the path for the EDS line scan. (i) The corresponding EDS mapping. (j) EDS line‐scan profiles of Ni and Pt elements in selected region. (k) XRD patterns of PtNi after different CV cycles.

EDS mapping reveals only a minor amount of Ni residue (21.5%) within the Pt skeleton, compared to the initial state (93.6%), demonstrating that most of the original Ni core had been leached out, leaving behind an alloyed structure with Pt. This final architecture combines the benefits of the alloy electronic effect with the high surface area and high atomic utilization efficiency of the nanocage structure, accounting for the performance improvement after activation [51]. This substantial Ni loss^ɛ^ was further corroborated by ICP‐OES analysis of the electrolyte, which detected progressively increasing dissolved Ni concentration with cycle number (Table S4), confirming that the EDS‐observed composition change reflects actual metal leaching.

For reference, the PtNi catalyst was subjected to the same procedural steps as the CV cycling without applying an external potential, which was maintained in an O_2_‐saturated 0.1 M HClO_4_ solution for a duration equivalent to 50 000 cycles (approximately 111 h). TEM analysis confirmed that the Ni@Pt core–shell structure remained intact (Figure S9), and EDS mapping revealed an atomic ratio consistent with the initial state of the PtNi catalyst (Table S5). These findings demonstrate that the dynamic in situ reconstruction of PtNi during CV activation is driven by the applied potential rather than by chemical corrosion.

The PtNi catalyst after different CV cycles was removed from the electrode and transferred onto carbon paper for XRD characterization. The results are shown in Figure 3k, where a consistently observed peak at approximately 54.5° is attributed to the carbon paper substrate (Figure S10). The initial diffraction pattern of PtNi on carbon paper matches that of the pristine powder PtNi, confirming the Ni@Pt core‐shell structure observed by TEM. As CV cycling progresses, the Ni(111) peak continues to shift to lower angles with increasing asymmetry. This systematic shift signifies a gradual increase in the average lattice constant, providing strong evidence for the selective leaching of Ni during the activation process. After 10 000 cycles, a distinct and broadened diffraction peak emerges at 41.4°, which is assigned to a Pt‐rich alloy phase. This finding aligns perfectly with the well‐defined Pt skeleton observed by TEM at the same stage. The intensity of this broad peak further increases after 15 000 cycles. In conclusion, the activation process is essentially a dynamic self‐optimization involving “corrosion–dissolution–redeposition”, which drives the spontaneous reconstruction of the initial core‐shell structure into a thermodynamically more stable and catalytically superior nanocage structure.

### 2.3 Mechanistic Origin of the Enhanced ORR Performance

To complement our analysis of the structural evolution and to unravel the structure‐activity relationship underlying the performance enhancement of PtNi during CV activation, we employed in situ electrochemical ATR‐FTIR spectroscopy to directly probe the dynamic evolution of the catalyst's surface chemistry and reaction intermediates. Specifically, we tracked the PtNi catalyst from its initial state through 1000, 5000, 10 000, and 15 000 CV cycles in both Ar‐ and O_2_‐saturated solutions, thereby elucidating the dynamic evolution of the solid‐liquid interface.

Figure 4a shows the in situ ATR‐FTIR spectrum of the initial PtNi measured in an Ar‐saturated solution. Characteristic vibrational features include the H─O─H bending mode at 1640 cm^−1^ and O─H stretching modes at 3612 and 3200 cm^−1^ [52, 53]. The sharp peak at 3612 cm^−1^ indicates that the interfacial water at the positively charged electrode surface adopts an ice‐like structure with non‐hydrogen‐bonded O─H [51, 54, 55, 56, 57]. In contrast, the ATR‐FTIR spectrum of the initial PtNi measured in an O_2_‐saturated solution reveals new features (Figure 4b): a band appears at approximately 1430 cm^−1^, attributed to the O─O stretching vibration of adsorbed O_2_. Concurrently, the intensity of the O─H bending vibration at 1640 cm^−1^ is significantly enhanced compared to that in the Ar‐saturated solution, indicating the generation of H_2_O on the surface due to the ORR [58, 59]. Notably, the O─H stretching envelope (3200–3400 cm^−1^) exhibits a pronounced broadening, suggesting a significant reorganization of interfacial water under reaction conditions [60].

**FIGURE 4:**
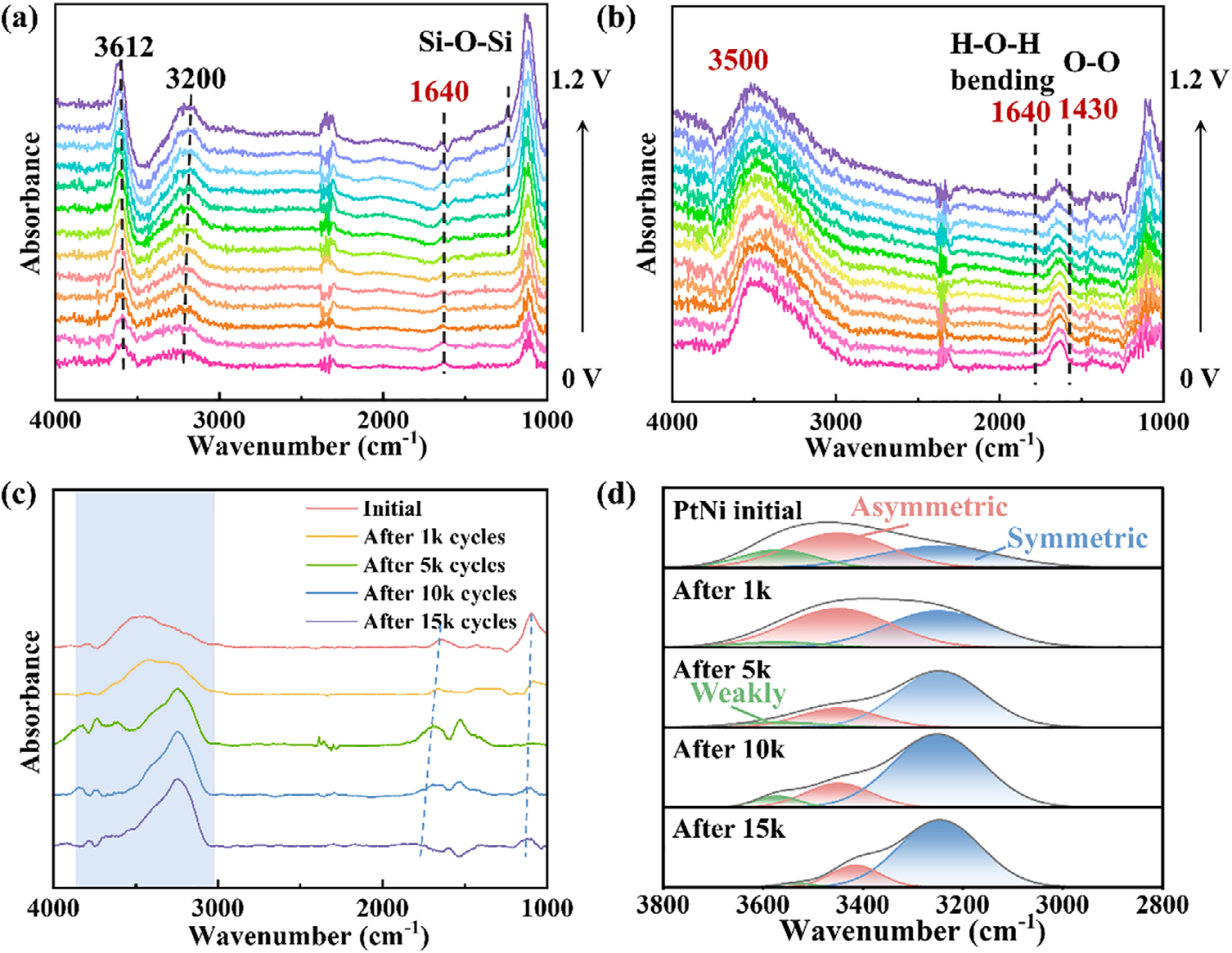
In situ ATR‐FTIR spectra of PtNi in (a) Ar‐ and (b) O_2_‐saturated 0.1 M HClO_4_. (c) In situ ATR‐FTIR spectra of PtNi at 0.7 V (vs. RHE) in O_2_‐saturated 0.1 M HClO_4_ after different CV cycles. (d) Deconvolution of the O─H stretching peak at 0.7 V into three components: green, weakly H‐bond; pink, asymmetric H‐bond; and blue, symmetric H‐bond.

As we track the evolution of interfacial water accompanying the reconstruction of the catalyst during CV activation (Figure 4c and Figure S11), a more gradual evolution unfolds in the 3000–3600 cm^−1^ region associated with surface O─H vibrations. Although changes in the local electric field, adsorbate coverage, and potential‐dependent water orientation may also influence the O─H band profile, the systematic redistribution among the literature‐assigned components under otherwise identical measurement conditions is consistent with an overall shift toward a more strongly hydrogen‐bonded interfacial water environment [52, 61]. Following previous in situ ATR‐FTIR studies [43, 60, 62], the O─H stretching envelope was deconvoluted into three components, corresponding to different hydrogen‐bonding environments: a weak hydrogen bond at 3570 cm^−1^, an asymmetric hydrogen bond at 3450 cm^−1^, and a symmetric (strong) hydrogen bond at 3270 cm^−1^ (Figure 4d and Table S6).

Quantitative integration (Table 1) reveals that as the number of CV cycles increases, the proportion of the symmetric hydrogen bond—indicative of a stronger bonding environment—progressively increases, while that of the weak hydrogen bond decreases. This evolution indicates a shift toward a more strongly hydrogen‐bonded interfacial water environment and occurs concurrently with the enhancement in ORR activity. To further examine the influence of surface reconstruction on interfacial water organization, explicit‐water AIMD simulations were performed for PtNi(111) and PtNi(211)‐D (Figure S12 and Table S7). The reconstructed PtNi(211)‐D surface exhibits a higher hydrogen‐bond coordination number and a larger population of short, near‐linear hydrogen‐bond configurations, together with shortened H···O and O···O distances [43, 60, 63], supporting a more connected interfacial hydrogen‐bond network consistent with the in situ infrared results.

**TABLE 1:** Hydrogen bond proportions derived from deconvolution analysis of the O─H stretching peak for PtNi at 0.7 V (vs. RHE) after different numbers of CV cycles, based on in situ ATR‐FTIR spectra.

Stage of PtNi	Symmetric H‐bond	Asymmetric H‐bond	Weakly H‐bond
Initial	35.0%	47.0%	18.0%
After 1k cycles	45.8%	48.7%	5.5%
After 5k cycles	72.1%	21.7%	6.2%
After 10k cycles	76.2%	18.3%	5.5%
After 15k cycles	81.9%	16.1%	2.0%

Previous studies have suggested that interfacial water serves as the proton donor in acid electrolytes. An efficient, strongly hydrogen‐bonded network facilitates proton tunnelling, whereas a deficient one severely impedes proton‐coupled electron transfer, leading to low activity [43, 44, 45]. The consequent facilitation of this step is directly evidenced by the concurrent attenuation of the OOH intermediate signal (∼1240 cm^−1^, Figure 4c), which confirms the accelerated consumption of this key intermediate [53, 59].

A coherent mechanism of the CV activation emerges from correlating our structural and spectroscopic data. The structural evolution depicted in Figure 5a—directly supported by TEM and XRD—demonstrates that the potential‐driven leaching of Ni and redistribution of Pt collectively drive the transformation of the PtNi catalyst from an initial core‐shell to a nanocage architecture. This structural reconstruction is not merely a morphological change; it is accompanied by the reorganization of the local electrochemical environment and interfacial water network. Based on previous reports showing that interfacial cations can reorganize hydrogen‐bonded water network [60], one plausible speculation is that transient enrichment of metal ionic species (Pt^2+^, Ni^2+^) released during leaching in the interfacial region could contribute to the observed increase in strongly hydrogen‐bonded water. However, we emphasize that this interpretation remains speculative and requires further experimental validation. Overall, these observations support a self‐optimization mechanism in which potential‐driven catalyst reconstruction is coupled with the reorganization of the interfacial water network, thereby creating a more favorable environment for proton transfer and contributing to the enhanced ORR performance. This coordinated evolution of the solid catalyst and liquid interface represents the synergistic solid–liquid dynamic coupling central to the proposed mechanism.

**FIGURE 5:**
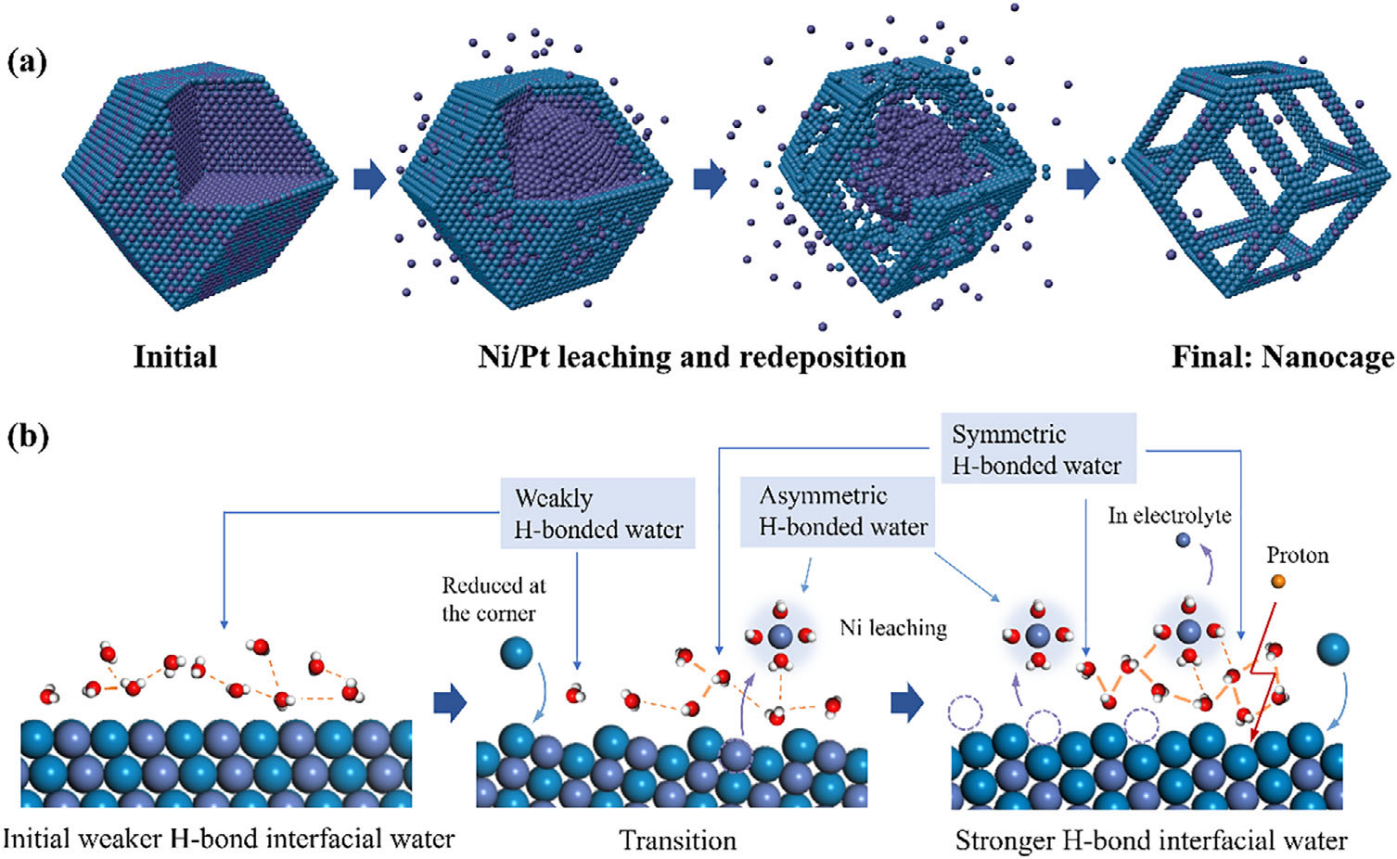
(a) Schematic illustration of the structural evolution of the PtNi catalyst during the CV activation process. (b) Schematic illustration of the dynamic surface reconstruction‐induced formation of a strengthened hydrogen‐bond network at the catalyst‐electrolyte interface. Color code: Pt, blue; Ni, purple; O, red; H, white; Proton: orange. Hydrogen bonds are represented as dashed lines for asymmetric/weak bonds and solid lines for strong bonds.

To unravel the atomic‐scale mechanism of performance enhancement driven by the dynamic in situ reconstruction of PtNi catalysts during CV activation, we employed density functional (DFT) calculations of the ORR on a series of experimentally motivated surface models. Quasi‐in situ structural characterization and compositional analysis indicate progressive Ni leaching, surface Pt enrichment, and the formation of an edge‐rich nanocage framework during CV activation. Based on these observations, four representative models were constructed (Figure 6a–d and Figures S13–S16): PtNi(111), representing the initial, inactivated alloy surface; PtNi(211), simulating a high‐index surface exposed during reconstruction; PtNi(211)‐Pt, representing a Pt‐enriched step‐edge environment; and PtNi(211)‐D, in which a surface Pt vacancy was introduced at the Pt‐enriched edge to represent a low‐coordination defect site. These models capture the representative surface motifs inferred from the experimentally observed reconstruction rather than constituting a direct atomistic trajectory of the transformation.

**FIGURE 6:**
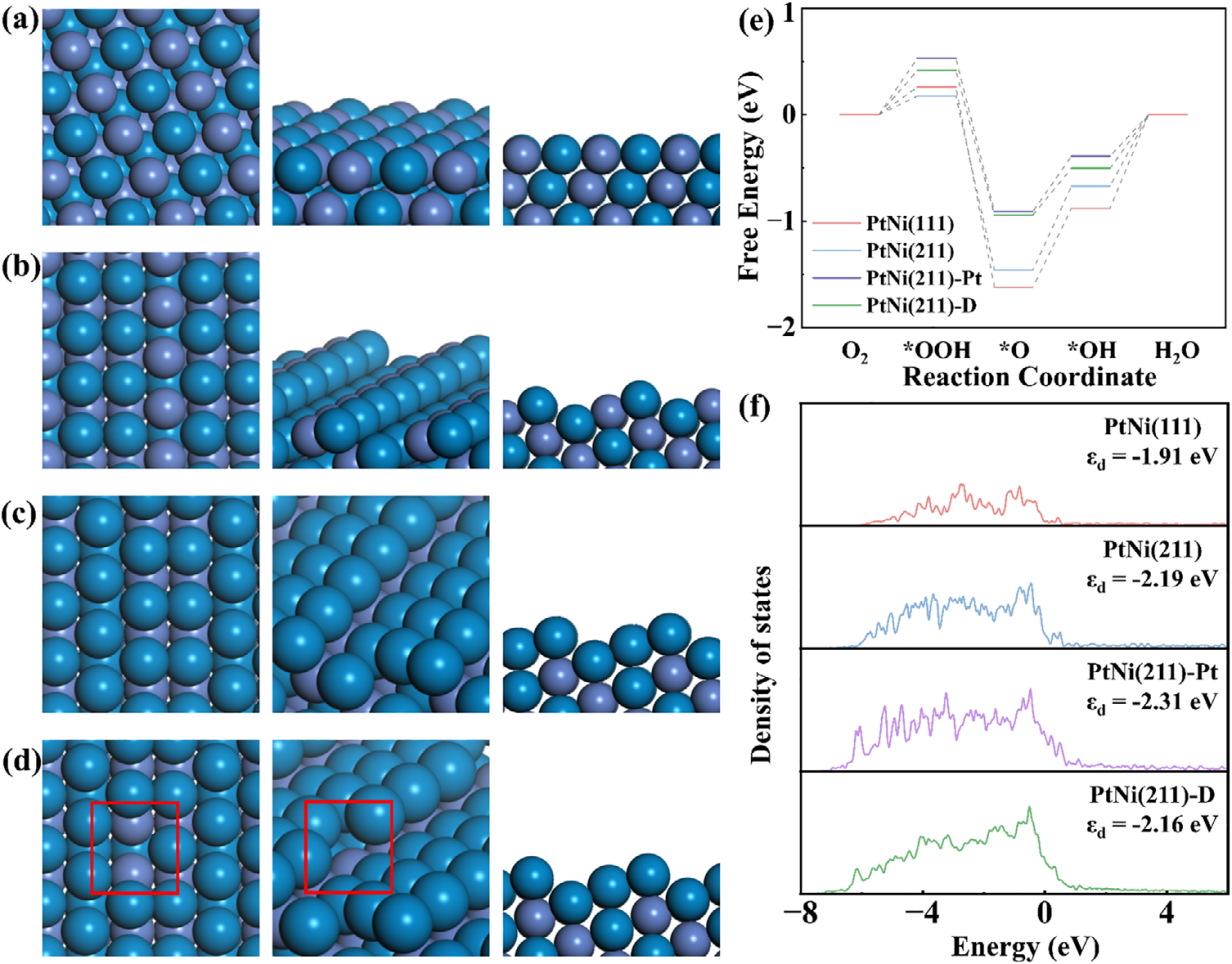
Optimized DFT models of (a) PtNi(111), (b) PtNi(211), (c) PtNi(211)‐Pt and (d) PtNi(211)‐D. Color code: Pt, blue; Ni, purple. (e) Free energy diagrams and (f) d‐band centers of PtNi(111), PtNi(211), PtNi(211)‐Pt and PtNi(211)‐D.

The calculated ORR overpotentials decrease from 0.86 V for PtNi(111) to 0.78 V for PtNi(211), 0.60 V for PtNi(211)‐Pt, and 0.49 V for PtNi(211)‐D (Figure 6e). This trend supports the experimental observation that potential cycling progressively generates more favorable ORR surface motifs. In particular, the low overpotential of PtNi(211)‐D identifies the Pt‐rich defective edge as a plausible highly active site formed during reconstruction [64, 65, 66, 67, 68].

To clarify the electronic origin of these differences, the average d‐band centers of the surface Pt atoms were compared with the adsorption free energies of OOH*, O*, and OH* (Table S8). The d‐band center shifts downward from −1.91 eV for PtNi(111) to −2.19 eV for PtNi(211) and −2.31 eV for PtNi(211)‐Pt. Correspondingly, the adsorption free energies of O* increase from 0.86 to 1.03 and 1.29 eV, while those of OH* increase from 0.37 to 0.58 and 0.66 eV, indicating progressively weaker stabilization of these intermediates as the d‐band center moves away from the Fermi level [69, 70]. In contrast, the OOH* adsorption free energies vary from 3.99 to 3.91 and 3.96 eV, showing a less monotonic dependence because the molecular OOH* intermediate is more sensitive to the local adsorption configuration.

For PtNi(211)‐D, the average surface‐Pt d‐band center shifts upward to −2.16 eV relative to PtNi(211)‐Pt, whereas the adsorption free energies of OOH*, O*, and OH* further increase to 4.15, 1.54, and 0.74 eV, respectively. This deviation from simple d‐band‐center scaling indicates that adsorption at the defective edge is additionally governed by its local coordination environment and adsorption geometry [71]. The superior calculated ORR activity of PtNi(211)‐D therefore does not originate from a uniform change in adsorption strength. Instead, the potential thresholds for OOH* formation, O* protonation, and OH* removal are balanced at approximately 0.77, 0.80, and 0.74 V, respectively. The resulting limiting potential of approximately 0.74 V corresponds to the lowest theoretical overpotential of approximately 0.50 V. These results suggest that the reconstructed defective edge provides a favorable energetic balance among the successive ORR intermediates.

## 3 Conclusion

This study demonstrates that potential cycling can drive the dynamic reconstruction of PtNi catalysts toward an enhanced and subsequently stabilized catalytic state. Quasi‐in situ TEM and XRD, together with ICP‐OES analysis, reveal that rapid initial Ni leaching and Pt surface redistribution transform the polyhedral core–shell particles into Pt‐rich nanocages. This reconstruction results in a 2.3‐fold increase in mass activity at 0.90 V vs. RHE after 10k cycles. The substantially slower metal release during subsequent cycling and the preserved nanocage morphology after 50k cycles further indicate the gradual stabilization of the reconstructed catalyst.

The activity enhancement is associated with the concurrent evolution of the catalyst structure and its interfacial water environment. The formation of the porous nanocage increases the electrochemically accessible surface area and generates low‐coordination Pt sites, while DFT calculations indicate more favorable ORR energetics on the reconstructed defective‐edge structure. Concurrently, in situ ATR‐FTIR spectroscopy reveals an increased contribution of strongly hydrogen‐bonded interfacial water, and explicit‐water AIMD simulations further show that the reconstructed surface supports a more connected and geometrically compact hydrogen‐bond network. These results indicate that the enhanced ORR performance arises from the coupled optimization of the active surface and its surrounding interfacial environment, rather than from morphological evolution alone.

Overall, these findings establish an “activation–reconstruction–optimization” mechanism in which electrochemical activation induces an initially rapid and subsequently self‐limiting reconstruction. This coupled catalyst–solvent evolution extends established static structure–activity principles by introducing a dynamic interfacial dimension into the understanding and design of electrocatalysts. Future work integrating single‐cell fuel‐cell testing will be important for determining how the observed self‐optimization behavior translates from the well‐controlled half‐cell environment to practical device operation.

## Author Contributions

**Weizhi Wang**: conceptualization, methodology, software, data curation, investigation, validation, Writing – original draft. **Yingying Cai**: methodology, software, investigation. **Pengfei Tian**: conceptualization, methodology, validation, formal analysis, supervision, funding acquisition, project administration, writing – review and editing. **Jing Xu**: conceptualization, methodology, supervision, project administration, writing – review and editing. **Mingliang Zhu**: methodology, funding acquisition, resources, formal analysis. **Fuzhen Xuan**: supervision, funding acquisition, project administration, writing – review and editing.

## Conflicts of Interest

The authors declare no conflicts of interest.

## Supporting information




**Supporting File**: advs78749‐sup‐0001‐SuppMat.docx.

## Data Availability

The data that support the findings of this study are available from the corresponding author upon reasonable request.
