# Supplementary material for: Dynamic Self‐Optimization of PtNi Nanocatalysts Through the Concurrent Evolution of Active Sites and Interfacial Environment During Reaction
Source: Adv Sci (Weinh). 2026 Nov 11:e78749. Online ahead of print. doi: 10.1002/advs.78749 (PMC13755359; doi:10.1002/advs.78749)
Supplement: Supplementary file 1 [file ADVS-9999-e78749-s001.docx]

Supporting Information

Dynamic Self-Optimization of PtNi Nanocatalysts through the Concurrent Evolution of Active Sites and Interfacial Environment During Reaction

Weizhi Wang^1^, Yingying Cai^1^, Pengfei Tian^2,^*, Jing Xu^1,*^, Mingliang Zhu^2^, Fuzhen Xuan^2,^*

**Contents**

**1. Experimental Section**

**2. Computational Methods and Models**

**3. Supplementary Figures**

**4. Supplementary Tables**

**1. Experimental Section**

*Sample Preparation*: The PtNi catalyst was prepared through a high-temperature reduction route. In a typical procedure, 10 mL of oleylamine was mixed with 4 mL of a H_2_PtCl_4_·6H_2_O solution (0.1 g/mL) and 7 mL of a Ni(NO_3_)_2_·6H_2_O solution (0.1g/mL). The mixture was stirred at 120 ^o^C for 1 h until complete evaporation of water, resulting in a transparent solution denoted as the precursor solution. Separately, 50 mL of oleylamine was heated to 160 ^o^C under an Ar atmosphere, maintained at this temperature for 1 h, and then further heated to 230 ^o^C. At this temperature, 5 mL of precursor solution was rapidly injected into the reaction flask. The mixture was stirred for 40 min before the reaction was quenched. The resulting product was collected by centrifugation and washed three times with a mixture of ethanol and n-hexane. The washed product was then dispersed in 10 mL of n-hexane by ultrasonication, followed by the addition of 30 mg of carbon black and further ultrasonication for 1 h. After three additional washing-centrifugation cycles with ethanol and n-hexane, the final solid was dried at 60 ^o^C overnight and ground into a black powder, yielding the PtNi catalyst.

*Measurements and Characterizations*: Powder X-ray diffraction (XRD) patterns were collected using a Rigaku D/max 2550 X-ray diffractometer with Cu Kα radiation. The system was set to 40 kV and 100 mA during data acquisition. Data were collected in a continuous scan mode over the 2θ range of 10° to 80° with a step size of 0.02° and a scan rate of 10°/min. For the ex-situ measurements on power samples, the as-synthesized PtNi catalyst was directly mounted and leveled in the sample holder. For quasi-in situ measurements, the working electrode was promptly removed from the electrochemical cell after treatment at the target potential, thoroughly washed with copious amounts of ultrapure water, and blow-dried in a gentle stream of N_2_. The catalyst layer was then carefully scraped off and dispersed in ethanol by sonication. Drops of the resulting suspension were applied to clean carbon paper substrates, dried under an inert atmosphere, and immediately subjected to XRD analysis to minimize atmospheric exposure and preserve the catalyst structure along with any reaction intermediates.

X-ray photoelectron spectroscopy (XPS) characterization was conducted using a Thermo Scientific K-Alpha spectrometer equipped with a monochromatic Al Kα source (1486.6 eV), operated at 12 kV and 6 mA. During data acquisition, the pressure in the analysis chamber was maintained below 5.0 × 10^-7^ mbar, and a 400 μm X-ray spot was employed. Both survey spectra (pass energy: 100 eV; step size: 1 eV) and high-resolution regional scans (pass energy: 50 eV; step size: 0.1 eV) were collected. Energy calibration of the collected spectra was achieved by setting the adventitious carbon C 1s signal to a binding energy of 284.8 eV.

Inductively coupled plasma optical emission spectrometry (ICP-OES) was used to analyze the elemental mass fractions. Measurements were performed on an Agilent 5800 VDV spectrometer, which offers a wavelength range of 167 ~ 785 nm and a resolution of <0.007 nm. For analysis, the solid catalysts were fully dissolved in aqua regia.

Transmission electron microscopy (TEM) analysis was performed using a ThermoFisher Talos F200X electron microscope operating at an acceleration voltage of 200 kV. For the as-synthesized PtNi powder catalyst, the sample was directly dispersed in ethanol via ultrasonication, and the resulting suspension was drop-cast onto lacey carbon-coated copper grids. The quasi in-situ TEM sample were prepared as the same with the quasi-in-situ XRD samples to preserve electrochemical history. Specifically, the catalyst powder was harvested from the electrode, immediately transferred and dispersed in ethanol, and subsequently drop-coated onto carbon-coated copper grids.

In situ electrochemical attenuated total reflection Fourier Transform Infrared Spectroscopy (ATR-FTIR) was conducted using a PerkinElmer FTIR spectrometer equipped with a liquid nitrogen-cooled mercury cadmium telluride (MCT) detector. The working electrode was fabricated on silicon ATR prism, onto which a gold nanofilm was chemically deposited to serve as both a conductive layer and an IR reflector prior to catalyst loading. The electrochemical setup was completed with a platinum counter electrode and an Ag/AgCl (saturated KCl solution) reference electrode. IR spectra were acquired with 64 scans at resolution of 4 cm^-1^. Critically, the activation and measurement parameters used during the in-situ ATR-FTIR analysis were maintained identical to those used in rotating disk electrode measurements, ensuring experimental consistency across different techniques.

*Electrochemical measurement*: All electrochemical measurements were performed at 25℃ with a standard three-electrode setup. A rotating disk electrode (RDE) featuring a glassy carbon disk of 5 mm in diameter, covered with a thin catalytic layer, served as the working electrode. A platinum wire and a saturated Ag/AgCl (in KCl) electrode were used as the counter and reference electrodes, respectively. All reported potentials in this study were calibrated to the reversible hydrogen electrode (RHE) reference. The uncompensated solution resistance (Ru) was determined via the instrument’s built-in iR-compensation test, and 85% real-time iR compensation was applied using the positive-feedback function of a CHI 650e workstation.. The same compensation procedure was consistently applied at all cycling stages.

To prepare the catalyst ink, 3 mg of PtNi catalyst was blended with 2 mg carbon black, 500 μL of isopropanol, 300 μL of ethanol and 20 μL of 5 wt% Nafion solution, followed by ultrasonic dispersion. A 6 μL aliquot of the resulting ink was then deposited onto the polished RDE surface, forming a homogeneous catalyst film.

Cyclic voltammetry (CV) and linear sweep voltammetry (LSV) were conducted in an O_2_-saturated 0.1 M HClO_4_ electrolyte. The polarization curves for the ORR were recorded in the potential range of 0-1.2 V (vs. RHE) at a scan rate of 10 mV/s and a rotation rate of 1600 rpm. And potential activation cycles were performed using the same potential window as CV and LSV, with a scan rate of 100 mV/s. The electrochemical active surface area (ECSA) was determined by the hydrogen underpotential deposition (HUPD) method. CV curves were measured in an Ar-saturated 0.1 M HClO_4_ solution, and the ECSA was calculated from the integrated charge in the hydrogen adsorption/desorption region using a standard charge density of 210 μC/cm^2^.^[1]^ Accelerated durability tests (ADT) were performed by applying potential cycles between 0.6 and 1.0 V (vs. RHE) at a scan rate of 100 mV/s in an O₂-saturated 0.1 M HClO_4_ solution.

To ensure statistical reliability, all electrochemical measurements were repeated at least three times independently under identical conditions, and the data are presented as mean ± standard deviation (SD). The corresponding error bars are included in the figures.

The electron transfer number (n) and kinetic current density (j_k_) were determined through the Koutecky-Levich (K-L) equation calculation:

$$\frac{1}{j}=\frac{1}{j_{k}}+\frac{1}{j_{d}}=\frac{1}{j_{k}}+\frac{1}{B\omega^{1/2}}$$

$$B=0.2nF{(D_{o})}^{2/3}\upsilon^{{-1}/6}C_{O}$$

Where j signifies the experimentally obtained current density, which is governed by its kinetic current density (j_k_) and diffusion-limiting current density (j_d_).^[2]^The other terms are physical constant: F is the Faraday constant, Co is the oxygen solubility (1.26×10^-3^ mol/L), Do is the diffusion coefficient of O_2_ (1.9×10^-5^ cm^2^/s), and *ν* is the kinetic viscosity of the electrolyte (0.01 cm^2^/s). As the rotating speed is expressed in rpm, a pre-factor of 0.2 was used.^[3-5]^ The constant B was calculated using the provided physical constants, and the electron transfer number (n) was subsequently calculated.

**2. Computational Details**

Theoretical calculations based on density functional theory (DFT) were conducted using the Vienna ab-initio simulation package (VASP),^[6, 7]^ with computational parameters consistent with those described in previous studies.^[8-13]^ To model the catalyst evolution, we constructed a series of four-layer slab models. The initial PtNi(111) model adopted a 4×4 supercell, with reciprocal space integration performed using 3×3×1 Gamma-centered k-points. The PtNi(211) alloy framework model was constructed with a commensurate 4×4 supercell, employing a 2×2×1 k-point mesh. The PtNi(211)-Pt model was subsequently created by replacing all surface atoms of the PtNi(211) model with Pt atoms. Finally, the PtNi(211)-D model was generated by removing one surface Pt atom from the PtNi(211)-Pt model. The resulting PtNi(211)-D slab contains 47 Pt and 16 Ni atoms, corresponding to an overall Ni content of approximately 25.4 at.%, reasonably close to the experimentally measured value of 21.5 at.% after 10k cycles. The specific vacancy site was determined by comparing the formation energies of several candidate configurations and selecting the most stable one. A 12 Å vacuum space was incorporated along the direction normal to all surface slabs to eliminate artificial interactions between their periodic replicas. The electrochemical reactions were assessed using the computational hydrogen electrode (CHE) model.^[14, 15]^

AIMD simulations were performed using CP2K 2026.2 with the RPBE-D3 functional, DZVP-MOLOPT-SR-GTH basis sets, GTH pseudopotentials, and an auxiliary plane-wave cutoff of 400 Ry. Explicit water layers containing 39 and 52 H₂O molecules were added to the optimized PtNi(111) and PtNi(211)-D slabs, respectively. The bottom two metal layers were fixed, while all other atoms were allowed to move. NVT simulations were conducted at 300 K using a CSVR thermostat with a time constant of 100 fs and a timestep of 2.0 fs. After 5.0 ps of equilibration, a 1.6 ps production trajectory was collected, with coordinates recorded every 20 fs. Interfacial water molecules were identified by an O–metal distance below 4.0 Å. Hydrogen bonds were defined using O···O < 3.5 Å, H···O < 2.5 Å, and O–H···O > 150°, while short, near-linear configurations were identified using H···O < 2.0 Å and O–H···O > 165°. Trajectory-internal uncertainties were estimated using 4000 block-bootstrap resamples over eight 0.2 ps blocks.

**3. Supplementary Figures**

**Figure S1.** XPS spectra of the initial PtNi catalyst: (a) Pt 4f, (b) Ni 2p, and (c) survey spectrum.

**Figure S2.** (a) The ORR polarization curves of PtNi at different rotation speeds. (b) K-L plots of PtNi at different potentials.

**Figure S3.** ORR polarization curves recorded for PtNi catalysts with varying Pt/Ni atomic ratios at the initial state and after 1k, 5k and 10k cycles in O_2_-saturated 0.1 M HClO_4_ solution. Pt_1_Ni_9_ is the catalyst discussed in the study.

**Figure S4.** (a) HAADF-STEM of PtNi at the initial. (b) Overlay of the corresponding EDS elemental mapping of Pt and Ni. Individual elemental maps of (c) Ni and (d) Pt.

**Figure S5.** (a) HAADF-STEM of PtNi after 1,000 cycles. (b) Overlay of the corresponding EDS elemental mapping of Pt and Ni. Individual elemental maps of (c) Ni and (d) Pt.

**Figure S6.** HAADF-STEM images of the PtNi catalyst after 5,000 cycles at (a) low, (b) medium, and (c) high magnification.

**Figure S7.** (a) HAADF-STEM of PtNi after 10,000 cycles. (b) Overlay of the corresponding EDS elemental mapping of Pt and Ni. (c) High-resolution HAADF-STEM image of PtNi after 10,000 cycles. Individual elemental maps of (d) Ni and (e) Pt. (f) The corresponding FFT pattern of the red region in (c).

**Figure S8.** Morphological and elemental characterization of PtNi catalyst after 50k cycles: (a, e) TEM image, (b, f) HAADF-STEM image. Corresponding EDS elemental mapping showing the distribution: (c, g) Ni, and (d, h) Pt.

**Figure S9.** Morphological and elemental characterization of PtNi after immersion in 0.1 M HClO_4_ for a duration equivalent to 50,000 CV cycles: (a) TEM image, (b) HAADF-STEM image. Corresponding EDS elemental mapping showing the distribution: (c) the overlay, (d) Ni, and (e) Pt.

**Figure S10.** The XRD pattern of carbon paper substrate with carbon black.

**Figure S11.** The in-situ ATR-FTIR of PtNi after (a) 1,000 cycles, (b) 5,000 cycles, (c) 10,000 cycles and (d) 15,000 cycles in 0.1 M HClO_4_ O_2_-saturated solution.

**Figure S12.** AIMD analysis of the interfacial water structures on PtNi(111) and PtNi(211)-D. (a,b) Representative snapshots of interfacial water configurations, where the yellow dashed lines indicate hydrogen bonds identified using the same geometric criterion. (c) Distribution of hydrogen-bond coordination numbers among interfacial water molecules. Error bars represent the symmetric half-widths of the block-bootstrap confidence intervals. (d,e) Cumulative distribution functions of the H···O and O···O distances, respectively. Compared with PtNi(111), PtNi(211)-D exhibits fewer undercoordinated water molecules, increased populations of two- and three-coordinated water molecules, and shorter characteristic hydrogen-bond distances, indicating a more connected and geometrically compact interfacial hydrogen-bond network.

**Figure S13.** DFT-optimized adsorption configurations of oxygenated intermediates on the PtNi111. Top views and corresponding side views for O (a, d), OH (b, e), and OOH (c, f) adsorption.

**Figure S14.** DFT-optimized adsorption configurations of oxygenated intermediates on the PtNi(211). Top views and corresponding side views for O (a, d), OH (b, e), and OOH (c, f) adsorption.

**Figure S15.** DFT-optimized adsorption configurations of oxygenated intermediates on the PtNi(211)-Pt. Top views and corresponding side views for O (a, d), OH (b, e), and OOH (c, f) adsorption.

**Figure S16.** DFT-optimized adsorption configurations of oxygenated intermediates on the PtNi(211)-D. Top views and corresponding side views for O (a, d), OH (b, e), and OOH (c, f) adsorption.

**4. Supplementary Tables**

**Table S1.** MA, SA and ECSA of the PtNi catalyst at different stages of CV activation and after 50k cycles.

| Samples | MA(A/mg_Pt_) | SA(mA/cm^2^) | ECSA(m^2^/g) |
| --- | --- | --- | --- |
| Initial | 1.91 ± 0.33 | 3.23 ± 0.27 | 59.01 ± 6.83 |
| After 1k | 2.42 ± 0.12 | 2.33 ± 0.15 | 103.87 ± 2.98 |
| After 5k | 3.59 ± 0.07 | 2.35 ± 0.12 | 152.45 ± 5.08 |
| After 10k | 4.41 ± 0.54 | 3.02 ± 0.50 | 145.79 ± 7.71 |
| After 15k | 4.08± 0.51 | 2.87 ± 0.34 | 142.33 ± 3.44 |
| After 50k | 3.88± 0.47 | 2.76 ± 0.34 | 140.87 ±1.35 |

[a] The values are presented as mean ± standard deviation from three independent electrochemical measurements ((n=3)). MA and SA were determined at 0.90 V versus RHE.

**Table S2.** Comparison of ORR performance of PtNi-based catalysts in 0.1M HClO_4_ solution.

| Catalysts | E_1/2_(V vs. RHE) | MA (A/mg_Pt_) | SA (mA/cm^2^) | ADT | | Ref. |
| --- | --- | --- | --- | --- | --- | --- |
| PtNi | 0.924 | 4.41 | 3.02 | 50k | -3.8% | This work |
| TiAu@PtNi-400 | 0.93 | 2.72 | 5.26 | 20k | -<7% | [16] |
| PtNi@Ni-N-C | 0.942 | 0.91 | / | 30k | -21.4% | [17] |
| PtNi/CNF-1200 | 0.92 | ~0.33 | 0.52 | 5k | -15% | [18] |
| PtNi_3_@OMC-A | 0.907 | 3.23 | 2.11 | 10k | -20.8% | [19] |
| PtNi-BNCs/C | 0.93 | 5.16 | 3.52 | 50k | -1.3% | [20] |
| PtNi/Pd | 0.92 | 1.7 | 3.2 | 30k | -17.7% | [21] |

**Table S3.** Comparison of ORR performance of state-of-the-art Pt-based nanocage catalysts in 0.1M HClO_4_ solution.

| Catalysts | E_1/2_(V vs. RHE) | MA (A/mg_Pt_) | SA (mA/cm^2^) | ADT | | Ref. |
| --- | --- | --- | --- | --- | --- | --- |
| PtNi | 0.924 | 4.41 | 3.02 | 50k | -3.8% | This work |
| PtPdNiCuMnAu HENCs | 0.905 | 0.985 | 1.07 | 10k | -5.6% | [22] |
| Pt_3_Ni@Ni-N_4_-C | 0.93 | 1.92 | 2.65 | 30k | -1.2% | [23] |
| Pt_83_Ni_17_ BNCs AG | 0.94 | 1.95 | 3.55 | 20k | -17.4% | [24] |
| PtCoV-EPNF | 0.934 | 1.40 | 1.98 | 30k | -1.7% | [25] |
| h-PtNiCo/C | 0.932 | 1.03 | 2.75 | 5k | -13% | [26] |
| PtNi/h-Co-NC | 0.937 | 0.46 | 0.52 | 50k | -22.76% | [27] |
| Pt_39_Ir_10_Pd_11_ | / | 0.52 | 0.82 | 10k | -17.6% | [28] |
| Pt_3_PdNi NCs | / | 1.17 | 3.8 | 10k | -16% | [29] |

**Table S4.** ICP-OES results of Ni and Pt concentrations in the electrolyte after different cycles. ^[a]^

| Elements | Initial | After 1k | After 5k | After 10k | After 50k |
| --- | --- | --- | --- | --- | --- |
| Ni | n.d. | 0.062 | 0.080 | 0.088 | 0.110 |
| Pt | n.d. | n.d. | n.d. | n.d. | 0.063 |

[a] n.d. means not detected (below the ICP-OES detection limit).

**Table S5.** Evolution of the Ni/Pt atom ratio (from EDS mapping) of PtNi with different CV cycles compared with the acid-treated PtNi.

| Elements | Initial | After 1k | After 5k | After 10k | After 50k | Acid-treated |
| --- | --- | --- | --- | --- | --- | --- |
| Ni | 93.6 | 85.6 | 45.6 | 21.5 | 18.9 | 95.8 |
| Pt | 6.4 | 14.4 | 54.4 | 78.5 | 81.1 | 4.2 |

**Table S6.** Fitting parameters and uncertainties for the deconvolution of the O-H stretching region.

| CV cycles | Symmetric H-bond A±SE | Asymmetric H-bond A±SE | Weakly H-bond A±SE^[a]^ | Adj. R^2^ | RSS |
| --- | --- | --- | --- | --- | --- |
| Initial | 0.488±0.005 | 0.656±0.009 | 0.251±0.006 | 0.990 | 1.511E-5 |
| 1k | 0.728±0.004 | 0.773±0.007 | 0.088±0.004 | 0.995 | 0.897E-5 |
| 5k | 1.224±0.008 | 0.369±0.009 | 0.106±0.007 | 0.981 | 6.161E-5 |
| 10k | 1.646±0.010 | 0.395±0.011 | 0.118±0.007 | 0.985 | 6.384E-5 |
| 15k | 1.382±0.005 | 0.271±0.005 | 0.033±0.003 | 0.993 | 3.939E-5 |

[a]: A represents the integrated Gaussian peak area, and SE represents the standard error obtained from nonlinear least-squares fitting. All spectra were analyzed using identical fitting functions and constraints.

**Table S7.** Summary of AIMD-derived interfacial water and hydrogen-bonding descriptors on PtNi(111) and PtNi(211)-D.

| Descriptor | PtNi(111) | PtNi(211)-D |
| --- | --- | --- |
| Average H-bond coordination number | 1.60 | 1.84 |
| Short, near-linear configurations (%) | 12.5 | 19.5 |
| Mean H···O distance (Å) | 1.967 | 1.919 |
| Mean O···O distance (Å) | 2.921 | 2.877 |
| Interfacial water density (nm^-2^) | 9.81 | 10.07 |

**Table S8.** d-band centers (ε_d_) and adsorption energies of O* (∆*E*_O_), OH* (∆*E*_OH_) and OOH* (∆*E*_OOH_) intermediates ^[a]^

| Catalysts | ε_d_ (eV) | ∆E_O_ (eV) | ∆E_OH_ (eV) | ∆E_OOH_ (eV) |
| --- | --- | --- | --- | --- |
| PtNi(111) | -1.91 | 0.86 | 0.37 | 3.99 |
| PtNi(211) | -2.19 | 1.03 | 0.58 | 3.91 |
| PtNi(211)-Pt | -2.31 | 1.29 | 0.66 | 3.96 |
| PtNi(211)-D | -2.16 | 1.54 | 0.74 | 4.15 |

[a] The binding energies of O*, OH* and OOH* are defined as the reaction energies of the reactions: H_2_O + * → O* + H_2_ and H_2_O + * → OH* + 1/2H_2_, respectively.

**Reference**

[1] Rudi S, Cui C, Gan L, et al. Comparative Study of the Electrocatalytically Active Surface Areas (ECSAs) of Pt Alloy Nanoparticles Evaluated by Hupd and CO-stripping voltammetry[J]. Electrocatalysis. 2014, 5(4): 408-418.

[2] Ma R, Lin G, Zhou Y, et al. A review of oxygen reduction mechanisms for metal-free carbon-based electrocatalysts[J]. npj Computational Materials. 2019, 5(1).

[3] Allen J. Bard and Larry R. Faulkner, Electrochemical Methods: Fundamentals and Applications, New York: Wiley, 2001, 2nd ed[J]. Russian Journal of Electrochemistry. 2002, 38(12): 1364-1365.

[4] Guo L, Jiang W-J, Zhang Y, et al. Embedding Pt Nanocrystals in N-Doped Porous Carbon/Carbon Nanotubes toward Highly Stable Electrocatalysts for the Oxygen Reduction Reaction[J]. ACS Catalysis. 2015, 5(5): 2903-2909.

[5] Kim J, Lee S W, Carlton C, et al. Pt-Covered Multiwall Carbon Nanotubes for Oxygen Reduction in Fuel Cell Applications[J]. J Phys Chem Lett. 2011, 2(11): 1332-1336.

[6] Kresse G, Furthmüller J. Efficient iterative schemes for ab initio total-energy calculations using a plane-wave basis set[J]. Physical Review B. 1996, 54(16): 11169-11186.

[7] Kresse G, Furthmüller J. Efficiency of ab-initio total energy calculations for metals and semiconductors using a plane-wave basis set[J]. Computational Materials Science. 1996, 6(1): 15-50.

[8] Blöchl P E. Projector augmented-wave method[J]. Physical Review B. 1994, 50(24): 17953-17979.

[9] Kresse G, Joubert D. From ultrasoft pseudopotentials to the projector augmented-wave method[J]. Physical Review B. 1999, 59(3): 1758-1775.

[10] Perdew J P, Yue W. Accurate and simple density functional for the electronic exchange energy: Generalized gradient approximation[J]. Physical Review B. 1986, 33(12): 8800-8802.

[11] Perdew J P, Burke K, Ernzerhof M. Generalized Gradient Approximation Made Simple[J]. Physical Review Letters. 1996, 77(18): 3865-3868.

[12] Methfessel M, Paxton A T. High-precision sampling for Brillouin-zone integration in metals[J]. Physical Review B. 1989, 40(6): 3616-3621.

[13] Xing D, Dong L, Qi Y, et al. Ru-supported Cu nanowire catalyst enabling to suppress C–C coupling for high-selectivity ethylamine electrosynthesis[J]. AIChE Journal. 2024, 70(5): e18377.

[14] Peterson A A, Abild-Pedersen F, Studt F, et al. How copper catalyzes the electroreduction of carbon dioxide into hydrocarbon fuels[J]. Energy & Environmental Science. 2010, 3(9): 1311-1315.

[15] Nørskov J K, Rossmeisl J, Logadottir A, et al. Origin of the Overpotential for Oxygen Reduction at a Fuel-Cell Cathode[J]. The Journal of Physical Chemistry B. 2004, 108(46): 17886-17892.

[16] Fisseha G, Yu Y n, Lu S, et al. A novel core-shell nanostructure of Ti-Au nanocrystal with PtNi alloy skin: Enhancing the durability for oxygen reduction reaction[J]. Chinese Journal of Catalysis. 2024, 56(81-87.

[17] Zhou F, Ruan Y, Li F, et al. Integrating single Ni site and PtNi alloy on two-dimensional porous carbon nanosheet for efficient catalysis in fuel cell[J]. Nano Research. 2024, 17(8): 6916-6921.

[18] Zhuang Y, Yang J, Meng L, et al. Polyaniline-derived carbon nanofibers with a high graphitization degree loading ordered PtNi intermetallic nanoparticles for oxygen reduction reaction[J]. Industrial Chemistry & Materials. 2023, 1(3): 458-464.

[19] Wang K, Wang Y, Geng S, et al. High‐Temperature Confinement Synthesis of Supported Pt–Ni Nanoparticles for Efficiently Catalyzing Oxygen Reduction Reaction[J]. Advanced Functional Materials. 2022, 32(22).

[20] Tian X, Zhao X, Su Y-Q, et al. Engineering bunched Pt-Ni alloy nanocages for efficient oxygen reduction in practical fuel cells[J]. Science. 2019, 366(6467): 850-856.

[21] Zhao Y, Tao L, Dang W, et al. High-Indexed PtNi Alloy Skin Spiraled on Pd Nanowires for Highly Efficient Oxygen Reduction Reaction Catalysis[J]. Small. 2019, 15(17): e1900288.

[22] Liu Q, Kang H, Liu Y, et al. Overcoming the Catalytic Bucket Effect in Pt-based High-Entropy Nanocages Through Interface Defect and Strain Engineering[J]. Advanced Science. 2026, n/a(n/a): e76501.

[23] Yan W, Guo O, Xing Q, et al. Atomically Dispersed Ni–N4 Sites Assist Pt3Ni Nanocages with Pt Skin to Synergistically Enhance Oxygen Reduction Activity and Stability[J]. Small. 2023, 19(22): 2300200.

[24] Zheng Y, Petersen A S, Wan H, et al. Scalable and Controllable Synthesis of Pt-Ni Bunched-Nanocages Aerogels as Efficient Electrocatalysts for Oxygen Reduction Reaction[J]. Advanced Energy Materials. 2023, 13(20): 2204257.

[25] Zhao L, Zhu Z, Wang J, et al. Unlocking Proton Exchange Membrane Fuel Cell Performance with Porous PtCoV Alloy Catalysts[J]. Advanced Materials. 2025, 37(34): 2502457.

[26] Ma H, Zheng Z, Zhao H, et al. Trimetallic PtNiCo branched nanocages as efficient and durable bifunctional electrocatalysts towards oxygen reduction and methanol oxidation reactions[J]. Journal of Materials Chemistry A. 2021, 9(41): 23444-23450.

[27] Fu T, Ma K, Li S, et al. Tailoring mesoporous hollow carbon supports for PtNi nanoalloys: Co-Nx electronic regulation and mass transport enhancement in oxygen reduction reaction[J]. Nano Materials Science. 2026.

[28] Zhu J, Xie M, Chen Z, et al. Pt-Ir-Pd Trimetallic Nanocages as a Dual Catalyst for Efficient Oxygen Reduction and Evolution Reactions in Acidic Media[J]. Advanced Energy Materials. 2020, 10(16): 1904114.

[29] Kong F, Liu S, Li J, et al. Trimetallic Pt–Pd–Ni octahedral nanocages with subnanometer thick-wall towards high oxygen reduction reaction[J]. Nano Energy. 2019, 64(103890.
